# Delay in seeking treatment and associated factors among women with pelvic organ prolapse in Wolaita zone, Southern Ethiopia: Hospital based mixed method study

**DOI:** 10.1186/s12905-023-02346-8

**Published:** 2023-04-21

**Authors:** Adisu Worku Shitu, Ermias Wabeto Wana, Tadele Dana Darebo, Zewdu Berhanu Mune

**Affiliations:** 1 Offa woreda Health Office, Southern, Ethiopia; 2 Department of Public Health, Jinka University, Jinka, Ethiopia; 3 School of Public Health, Wolaita Sodo University, Wolaita, Ethiopia; 4 Boloso Bombe woreda Health Office, Southern, Ethiopia

**Keywords:** Fear, POP, Wolaita, Women, Stigma

## Abstract

**Background:**

Despite increasing efforts to improve access to diagnosis and treatment services, women with pelvic organ prolapse tend to stay at home for years before getting treatment. A large number of women, particularly in rural areas do not get early diagnosis and treatment, and they come with an advanced stage; but the reason for this appears unclear. Thus, this study aimed to determine the proportion and associated factors of delay in seeking treatment among women with pelvic organ prolapse (POP) in the Wolaita zone, Southern Ethiopia.

**Methods:**

A hospital-based cross-sectional study mixed with qualitative method was conducted in the Wolaita zone, Southern Ethiopia, from September to October 2021. A total of 422 women with pelvic organ prolapse were selected by systematic random sampling for quantitative data. The women recruited for in-depth interviews were purposefully selected until data saturation reaches via phenomenological study design. The quantitative data were collected by interviewer-administered questionnaire and reviewing clients’ medical records via an open data kit and then exported to and analyzed with a statistical package for social science version 25. The associated variables were determined by conducting a logistic regression model and are presented with the crude odds ratio (COR) and adjusted odds ratio (AOR) with their respective 95% confidence intervals (95%CI). All statistical tests were conducted at a 5% level of significance. The interview was first transcribed, coded, and categories and themes were generated by applying thematic analysis.

**Results:**

The study showed that 82.0% (95% CI: [74.8-89.3%]) of women with POP delayed seeking treatment. The delay to seek treatment was associated with a lack of support (AOR = 4.9; 95% CI [1.8–13.2]), low-income (AOR = 6.4; 95% CI [2.2–19.2]), fear of disclosure (AOR = 5.0; 95% CI [1.3–19.2]) and fear of social stigma (AOR = 4.6; 95% CI [1.5–14.2]). The reasons for the delay were feeling shameful to disclose, fear of stigma and cultural and spiritual beliefs.

**Conclusions:**

More than three-fourths of women with POP delayed seeking treatment, and improving support, improving the economy, raising awareness, and involving influential people in interventions are all necessary to avert it.

## Background

Pelvic Organ Prolapse (POP) is also termed utero-vaginal prolapse (UVP), and it is the descent of the pelvic organs into the vaginal wall from their normal anatomical position. This occurs due to defects in the supporting structures of the uterus and vagina named utero-sacral ligaments, the cardinal ligaments complex and the connective tissue of the urogenital membrane [1]. Its common clinical symptoms include the feeling of pressure and vaginal bulging, discomfort in the perineum, difficulty with lifting, sitting and walking, and urinary and bowel symptoms including incontinence and painful intercourse [1, 2]. According to the simplified Pelvic Organ Prolapse Quantification (S-POP) staging system, POP is anatomically staged from zero to four; stage IV is the maximal descent of the pelvic organ in which the entire extent of the vaginal mucosa is averted [3]. It is more common, more severe and disproportionally affects women living in low-income countries. Multiple parity, early marriage and childbirth, limited and poor obstetric care, significant physical burdens, and socio-cultural beliefs all increase their risk [4–6].

There are different scientifically proven POP treatment methods; lifestyle changes including losing weight, pelvic floor muscle training (PFMT), vaginal pessaries, surgery or a combination of treatments. The treatment decision often depends on the stage and severity of the condition. Early detection of prolapse is preferable as some conservative treatments like lifestyle changes and PFMT may prevent the aggravation of symptoms and reduce the need for surgical treatment [7–9]. The women with POP face numerous personal and interpersonal challenges affecting their life quality [10] and experience complications like pain in the pelvis, abdomen and lower back, pain during sexual intercourse, urinary urgency and leaking urine, constipation and difficulty in walking long or standing for a long time [11]. Although they experience many of those challenges affecting their life quality, only a few of them seek early professional help [12]; stayed 36.41 months [13] to 85.8 months [14] since the onset of the symptoms without getting care and treatment. The study carried out in southern Ethiopia witnessed that 84.6% of women on POP treatment delayed to start the treatment, and their delay was associated with a lack of support, low income and fear of social stigma [13].

Poverty, lack of awareness of the condition, spiritual beliefs and feeling shameful to disclose the condition were associated with delayed care seeking among the women [14–16]. Even though the treatment is available at hospitals and delivered free of charge, women with the condition found to be delayed to start the treatment. Thus, understanding the factors and reasons that really are associated with delay in seeking treatment for POP is very important to guide the intervention and facilitate the early treatment, but there is a significant scarcity of substantial evidence in Ethiopia. Therefore, this study aimed to identify the delay in seeking treatment for POP and the associated factors among women in Wolaita Zone, Southern Ethiopia.

## Methods

### Study area

The study was conducted in the Wolaita Zone: one of the 13 zones in the Southern Nations, Nationalities, and People’s Region (SNNPR), which had a total population of 2,161,842 with male to female ratio of one. According to the Zone health department report for 2020, there were about 68 health centers, five public primary hospitals, two private general hospitals (Sodo Christian and Dubo St. Mary hospitals) and one teaching and referral hospital. All of the health centers and primary hospitals conduct POP screening services and refer to Wolaita Sodo University comprehensive specialized hospital (WSUCSH), Sodo Christian hospital and Dubo St. Mary Catholic Primary Hospital for further diagnosis and treatment. The hospitals have a total of 186 beds providing obstetric and gynecologic beds service including POP operations. There were no specific beds dedicated for POP treatment, but unoccupied beds in gynecological wards used for treating POP patients. The maximum of nine women get treated for POP per day and the cost was covered by local government and non-governmental organizations during the study period.

### Study design and period

A hospital-based cross-sectional study supplemented with phenomenological study was conducted from September to December 2021.

### Source and study population

The source population was all women with pelvic organ prolapse admitted to the gynecological wards of WSUCSH, Sodo Christian and Dubo St. Mary hospitals for POP treatment during the data collection period, and the study population was all women with pelvic organ prolapse admitted to the treatment and selected to participate. Women with incomplete data were excluded from the study.

### Sample size determination

We used a single population proportion formula to calculate the sample size for quantitative data with the following assumptions: an expected 50% delay for treatment, a 5% significance level and a 5% error to tolerate. With this assumption, a sample size of 384 was computed. The sample sizes calculated for factors found to be lower than 384. After adding 10% to compensate for expected non-response, the final sample size became 422 and was used to determine both objectives. The in-depth interview was continued until the information saturates.

### Sampling procedure

The study was conducted at three hospitals providing POP treatment; WSUCSH, Sodo Christian and Dubo St. Mary. The study subjects required from each hospital were proportionally allocated based on the number of POP patients admitted to the gynecological wards of the respective hospitals. A total of 960 women with POP were registered at gynecological departments to get treated; 380, 320, and 260 women at WSUCSH, Sodo Christian hospital and Dubo St. Mary hospital, respectively. In this regard, a sample size of 167, 141, and 114 was allocated to WSUCSH, Sodo Christian hospital and Dubo St. Mary hospital, respectively. The respondents were recruited using a systematic random sampling technique among the women admitted to get treated for POP at each hospital with an interval of two. The first respondent was randomly selected from the first two patients, and the succeeding respondents were recruited by adding two to the respective level of admission. The reason for delay was further assessed by qualitative study, and the participants were purposively selected from the women delayed to get treated for POP. The interview with the women was continued until the information saturates.

### Data collection methods, instrument and measurement

For quantitative data, a structured questionnaire was adapted from previously conducted similar studies [13, 14, 17, 18] and translated into Amharic and back to English by fluent speakers of both languages. The questionnaire had sections to assess the socio-demographic characteristics of the household, obstetric and gynaecological history and current symptoms of uterine prolapse among the women, and social and cultural thoughts, perceptions and beliefs. The stages of pelvic organ prolapse were then confirmed by reviewing the patient’s card (record review) to ensure whether the women who reported symptomatic prolapse had anatomical prolapse or not.

Two midwives fluent in local (Wolaita) and Amharic languages and one senior midwife supervisor from each hospital were trained for data collection and collected the quantitative data with the Open Data Kit (ODK) application under close supervision by investigators. The pretesting of questionnaire was conducted at Gesuba Primary hospital among twenty women who were diagnosed with POP prior to the actual data collection. The pretested questionnaire was administered to women admitted to POP treatment in the local language (Wolaita) after training data collectors on the study overview, ways of approaching the women, communication, respecting the cultural norms of the women, the informed consent process, the administration of the questionnaire and confidentiality of the data. The collected data were checked for completeness and consistency, and a necessary correction was made accordingly. The qualitative data were collected by conducting an in-depth interview using a guide among purposively selected women who were delayed for at least 12 months before starting the treatment and diagnosed with an advanced stage of prolapse. The interview guide was adapted from works of literature [19, 20] and voices were recorded during an interview and short notes were taken when necessary from the twelve interviewees. The interviewees were triangulated with respect to age, residence and stage of the prolapse.

### Variables

The dependent variable was a delay in seeking treatment for pelvic organ prolapse, and the independent variables were age, marital status, residence, occupation, level of education, and income, perceived social stigma, women’s decision-making power, lack of awareness, fear of surgery, cultural thoughts and beliefs (cultural influence), fear of disclosure, access to transportation, availability and access to treatment centers, health care cost and attitude of care providers and psychosocial care and support.

### Operational definitions

#### Delay in seeking care/treatment for POP

Seeking care or treatment for a POP at 12 months or later after the onset of symptoms (for new patients) or recurrence of prolapse after prior intervention (for repeats) [14, 21].

#### Treatment for POP

Remedial action including non-surgical treatments (exercise, vaginal pessaries, diet, life style change) and surgical treatments.

#### Advanced stage prolapse

According to the Quantitative Pelvic Organ Prolapse (POP-Q) System, Stage III and IV pelvic organ prolapses are considered advanced stage prolapses [22, 23].

#### Low income

Earning less than or equal to 1200 Ethiopian birr per month (poverty line in Ethiopia) was considered low income [14, 24].

#### Lack of psychosocial support

Those women who delayed seeking treatments because they do not have a person who supports them financially, materially and emotionally [14, 19].

#### Lack of decision making power

Women who had willingness to get care but sought the permission of others including the husbands to go to the treatment.

#### Lack of awareness

Women did not know sign and symptoms, causes of POP and availability of treatment.

#### Fear of social stigma

A woman with POP who delayed seeking treatments due to perceived social discrimination [14, 25].

#### Cultural influence

Feeling shameful to show their sexual body parts to others and looking for local traditional remedies for POP.

### Data analysis

The quantitative data were exported and analyzed with SPSS version 25. A descriptive analysis was conducted to identify the proportion, and bivariate and multivariable logistic regression analyses were performed to determine the associated factors of delay in getting POP treatment.

The variables with a p-value less than 0.2 in the bi-variable analysis were considered candidates for multi-variable analysis. Multi-collinearity test was carried out to check whether the independent variables were interdependent or not at a variable inflation factor less than ten or a tolerance greater than 0.1, and we found no variables interdependent. The goodness of fit was assessed using the Hosmer and Lemeshow goodness test at a non-significant p-value; it was found to be 0.32. A multivariable logistic regression model was conducted to identify variables statistically significantly associated with the dependent variable at a p-value less than 0.05. We presented the findings with proportions and AORs with respective 95% CIs. The reasons for the delay in seeking POP treatment were further explored by conducting in-depth interviews with 12 women diagnosed with advanced-stage prolapse who stayed for at least 12 months after the onset of the symptoms to start treatment. The audio data and field notes transcribed and read thoroughly again and again to draft codes. After having codes in hand, categories and then themes were generated. Thematic analysis with open code software resulted in six categories and three themes. The result was written in narrative form quoting strong supporting ideas from the interviewees.

## Results

### Socio‑demographic characteristics

A total of 412 women with POP participated in the study, and the response rate was 97.6%. The women were 48.90 ± 6.91 (SD) years old on average. More than three quarters (78.2%) of them were married, and 53.9% had no formal education. The majority; 87.9%, were living in a rural areas (Table 1).

### The proportion of delays in seeking treatments

Out of 412 women participated in the study, the majority (82.1%; 95% CI = 74.8-89.3%) delayed 12 months or more to initiate either surgical or non-surgical treatment after they developed prolapse. The mean time duration for starting POP treatments after the onset of prolapse was 43.6 ± 22.5 months. More than one-half (60.9%) presented with stage III prolapse, while the remaining 30.1% and 8.9% sought treatment for stage II and IV POP, respectively.

**Table 1:** Socio-demographic characteristics of women with POP in Wolaita zone, 2021 (n = 412)

Variable	Frequency(n)	Percentage
**Age groups(in years)**		
35–44	110	27.0%
45–54	206	50.0%
55–64	88	21.4%
65 years and above	8	1.9%
**Marital status**		
Married	322	78.2%
Divorced	13	3.2%
Widowed	77	18.6%
**Educational status**		
Couldn’t read and write	222	53.9%
Literate	190	46.1%
**Residence**		
Rural	362	87.86%
Urban	50	12.1%
**Occupation**		
Housewife	331	80.3%
Governmental employer	19	4.6%
Merchant	50	12.1%
Others	12	3.0%
**Monthly income**		
> 1200 ETB	343	83.3%
≤ 1200 ETB	69	16.7%

### Reasons for delays in seeking treatment for POP

Out of 338 women with POP who delayed seeking the treatment, 95.2%, 93.5%, and 91.7% women complained of cultural influence, fear of disclosure, and low income as the reasons for the delayed treatment, respectively. More than three-quarters of the women (89.0%) mentioned fear of stigma as the reason for the delay, and 83.1% of them reported a lack of support as the reason for the delayed initiation of the treatment for POP (Table 2).

**Table 2:** Reasons for delayed treatment among women with POP in Wolaita zone (n = 338)

Variables	Frequency	Percent (%)
High health care cost	85	25.1%
Lack of access to treatment center	242	71.6%
Lack of transportation	243	71.9%
Lack of information	244	72.2%
Lack of support	281	83.1%
Fear of stigma	301	89.0%
Low income	310	91.7%
Fear of disclosure	316	93.5%
Culture influence	322	95.2%

Reasons for delay in seeking POP treatment were further assessed by conducting in-depth interviews with 12 women with advanced stages of prolapse and delayed getting treatment. All of the interviewees were married (Table 3).

**Table 3:** socio-demographic characteristics of the interviewees in Wolaita zone, 2021

Characteristics	Frequency	Percentage
**Age**		
40 years and above	12	100.0
**Occupation**		
Farmer and small scale business	12	100.0
**Educational status**		
Illiterate	8	66.7
Literate	4	33.3
**Marital status**		
Married	12	100.0

Fear of disclosure, fear of stigma and cultural thoughts were reasons for delayed treatment.

### Fear of disclosure

Among the interviewed women, 8 of 12 (67.0%) mentioned that the main reason for their delay was fear of disclosure to their husbands, family members, and neighbours. This is because reproductive system illness was considered a social taboo in their community, and it was thought to be a shame to discuss it with anyone else. “……………it is hard and just a difficult thing to explain to people most of the time, even to my husband and daughters… Most people do not want to hear about it. I become worried and depressed for myself on this condition and even cry for long times in the hidden place’’ (50 years old woman with advanced POP and stayed for five years with POP and not being treated). *‘*’……for me, it is sad to admit, but it may be another emotion…it is a shame. Even sometimes, I ask myself, what is the importance of telling other people if there is no solution,. I would hardly talk to anyone about it… you do not want to talk to someone who does not know about it or understand. You are ashamed to talk about it” (58 years old woman with POP and delayed for six years).

### Perceived stigma

Among the interviewed women, 10/12(84.0%) explained that their delay in getting treatment was directly or indirectly related to fear of stigma and discrimination from their family members, neighbours and other community members. They were not only worrying about the future but also facing stigma and discrimination due to disclosing their status.

“………………most of the time, I see women who get divorced without any tangible reason….when I realize the reason, it might be because of the conditions like this. I know some women who were chased away from their homes due to this kind of problem. I was very concerned that one day my husband might know or suspect that I have this problem and get divorced’’ (48 years old woman with stage III prolapse and delayed for five years).

‘’……………I had the decision to tell others about the condition and need some information regarding treatment options. But I worry that they might badly treat me, discriminate me and talk about me behind because I know that many women were victims of gossip’ (56 years old woman with POP and delayed for eight years).

‘’………….my husband is not concerned about my health even though he is currently living with his second wife, and he doesn’t know whether I am healthy or ill. One time when I told him about the issue, he insulted me and tortured me by stopping any help ….Eventually, he stopped to come to my home for a long time (46 years old woman with stage III prolapse and delayed for four years).

### Cultural influence and beliefs

Among the women interviewed, 8 of 12 (67.0%) stated that cultural beliefs and community practices prevented them from seeking treatment and receiving timely care. The women thought that the local remedies like massaging the abdomen and drinking the juice of plants gave relief and treated the condition. Others mentioned that spiritual healings like prayer and drinking holy water (spa) were better than visiting modern treatment facilities, and claimed that modern treatment facilities charge more and carry out exhaustive processes.

‘’…………in our society, it is believed that as a normal event for aged women and not a new event for women who bear many children. I also believed it as the sign of terminating childbearing and common around late ages of women. I tried to improve my health with a good nutrition and by managing my body, but I saw no changes’’ (57 years old, women with sage IV prolapse and delayed for six years). ‘’…………….from the very beginning, I believed that further medical care is needed when it advances and worsens….even some educated people simply talk about it; saying that surgery is done only for those who have massive prolapse, and I have been waiting for a long time until that occurs to me and in the meantime looking for God’’ (55 years old women with sage IV prolapse and delayed for six years).

### Factors associated with delay in seeking treatment for POP

In bivariate logistic regression analysis, nine variables (lack of support, no access to transportation, low income, lack of information, fear of disclosure, fear of stigma, cultural influence, perceived high health care cost and unavailability of treatment centers nearby) were included and found to be candidate variables for the multivariable logistic regression model. Then, after controlling the confounding effects in the multivariate logistic regression model, five variables (lack of support, low income, fear of disclosure, fear of stigma and perceived high health care cost) were statistically significantly associated with delayed treatments for the POP at a p-value less than 0.05. The odds of delay in seeking treatment for POP among women who lacked support were five times [AOR = 5.0; 95% CI (1.8–13.2)] more likely than their counterparts. The Women with lower income had six [AOR = 6.4; 95% CI (2.2–19.2)] times increased odds of delayed treatment than women with higher income. When compared to their counterparts, women who perceived social stigma were about five times [AOR = 4.7; 95% CI (1.5–14.2)] more likely to delay starting treatments. Women who did fear to disclose their status had five times [AOR = 5.0; 95% CI (1.3–19.2)] times increased odds of delay in seeking treatment than the women on the other side. Women who perceived high health care costs had 20.0% [AOR = 1.2; 95% CI (1.1–2.5)] increased odds of delay in seeking treatment as compared to the women on the other hand (Table 4).

**Table 4:** Bi-variable and multi- variable analysis of factors associated with a delay in seeking treatment for POP, Wolaita zone, SNNPR, Ethiopia, 20,121

Variables	Delayed treatment (n = 338)	Crude odds ratio (95%CI)	Adjusted odds ratio (95%CI)
Lack of support			
No	57(52.8%)	1	1
Yes	281(92.4%)	11.1(4.9–17.5)	5.0(1.8–13.2)**
Low income			
No	28(39.4%)	1	1
Yes	310(90.1%)	15.4(5.1–18.2)	6.4(2.2–19.2)**
Lack of transportation			
No	95(67.8%)	1	1
Yes	243(89.3%)	4.0(2.1–6.5)	1.72(0.6–4.8)
Fear of disclosure			
No	22(30.6%)	1	1
Yes	326(93.1%)	10.5(5.4–16.6)	5.0(1.3–19.2)**
Fear of stigma			
No	37(39.4%)	1	1
Yes	301(94.6%)	5.1(11.4–23.1)	4.6(1.5–14.2)**
Lack of information			
No	94(77.7%)	1	1
Yes	244(83.8%)	1.26(0.7–2.3)	2.4(0.9–6.1)
Unavailability of treatment center			
No	96(70.6%)	1	1
Yes	242(87.7%)	2.9(1.0-3.08)	0.4(0.1–1.2)
Perceived high health care cost			
No	105(60.3%)	1	1
Yes	223(66.0%)	2.8(1.1–6.6)	1.2(1.1–2.5)**

**variables statistically significantly associated with the dependent variable

## Discussion

The current study assessed the proportion and factors associated with delay in seeking treatment for POP in the Wolaita zone, southern Ethiopia. Among the women admitted to the hospitals to get treated for POP, 82.0% (CI = 78.3-85.7%) delayed seeking treatment, and the average length of delay was 43.6 ± 22.5 months. A lack of support, low income, fear of disclosing the condition, fear of stigma and perceived high health care costs were found to be statistically significant factors. Cultural influences and beliefs, fear of disclosing their condition to spouses, neighbours and health workers and lack of support were reasons for delayed treatment initiation among the women in the study area.

The study showed that 82.0% of women delayed seeking POP treatment with the mean length of delay of 62 ± 22.53 months. The proportion of delay is consistent with the evidence revealed by the study conducted in the northern part of Ethiopia, Gondar referral hospital in northwest Ethiopia; (82.6%) [14] and in selected general and referral hospitals in Southern Ethiopia; (84.6%) [13]. This indicates that the higher proportion of women with POP delay to start treatment in different settings of Ethiopia, and it may be a national problem. Thus, it calls for wider scope interventions and joint efforts to alleviate.

The proportion of delay revealed by the current study is higher than that observed in the United States of America (USA); 61.4% [21]. The possible explanation for the inconsistency may be the difference in parity, occupation (more than half of the studied women were employed in USA), improved access to health facilities, yearly home visits by health-care providers and improved educational status of the women in the USA (the study participants attained junior to graduate class education).

Women who lacked support were about five times more likely to delay getting treated for POP than their counterparts. The finding is consistent with the studies carried out at selected general and referral hospitals in Southern Ethiopia [13], Iran [26, 27] and middle eastern women [28]. This shows that social, economic and emotional support is important to enhance early treatment of POP among the women in the study area and elsewhere.

Women with a low income had 6.4 times increased odds of delay to start treatments for POP than the women on the other hand. This finding supports the study conducted at selected general and referral hospitals in Southern Ethiopia [13] and Gondar University Hospital, northwest Ethiopia [14, 16]. According to the current study, women who feared disclosing the condition were five times more likely to delay in seeking treatments than their counterparts. The finding was supported by the study conducted at selected general and referral hospitals in southern Ethiopia [13] and the studies conducted at Gondar, northwest Ethiopia, Iran and Netherlands [14, 16, 27, 29]. The odds of delay in seeking treatment among the women who perceived high health care costs were 20.0% higher than their counterparts. This was because women did not recognize that POP diagnosis and treatment are free of charge and these needs disseminating information on its basics, treatment options, treatment places and service related costs and keeping confidentiality of the patients.

The qualitative findings of the current study showed that fear of social stigma, fear disclosure and cultural influences and beliefs were the main reasons for the delay in seeking POP treatment; support the quantitative data. Because POP involves the most sensitive part of the body (genitalia), the women tend to hide it, and the finding is consistent with the evidence revealed by other studies: social stigma is the most explained barrier to seeking health-care and exposing problems even to close ones. Furthermore, because they are afraid of discrimination and rejection from others, they conceal the illness and only reveal it when it becomes severe and unbearable after a long period of time since they first notice symptoms. As a result, they delay for years and only present to hospitals when the problem has progressed to the point where surgical interventions or solutions are required [16, 17, 29, 30].

The current study showed that the perception of cultural healing and spiritual thinking (beliefs) toward POP in the community were mentioned as reasons for delayed treatment. In addition, women were unwilling to show problems related to their genitalia (sensitive body parts) because of the feeling of shame, which has a deep root in society’s culture. In addition to praying, they were used to perform remedies such as massaging the abdomen and drinking plant juice. This finding is consistent with the evidence from the study conducted in Nepal which identified that a ‘’culture of silence” restricted women from talking about pregnancy and related reproductive health problems like POP [17], Amhara regional state [14, 16] and southern Ethiopia [13].

### Limitations

Since this study was facility-based (hospital-based), it might miss the women who had no access to treatment, mainly in remote rural areas with long delays and advanced stages of prolapse. Due to its cross-sectional nature, it cannot show the temporal relationship between cause and effect. The time from the onset of the symptom until starting the treatment was self-reported; there might be miscounting of duration (oldies women count durations relative to events) that might exaggerate the delay time to start treatment; therefore, precaution is needed when interpreting.

## Conclusion and recommendations

The majority of the women with pelvic organ prolapse delayed seeking treatment in the study area. Their delay in getting treatment was associated with a lack of support, low income, fear of stigma, fear of disclosure, perceived high health care costs, and cultural influences and beliefs. To reduce the time between the onset of symptoms and receiving POP treatment among the women with the problem, improving awareness among women, early case finding, psycho-social support, involving religious and influential people, and economic empowerment are all recommended.

## Data Availability

The datasets analyzed during the current study are available in the Harvard Dataverse repository, 10.7910/DVN/FCSXYM .

## Abbreviations

AOR: Adjusted Odds Ratio
CI: Confidence Interval
COR: Crude Odds Ratio
ODK: Open Data Kit
POP: Pelvic Organ Prolapse
PFMT: Pelvic floor muscle training
RERC: Research Ethical Review Committee
SNNPR: Southern Nation’s Nationalities and People Region
SPSS: Statistical Software for Social Science
WSUCSH: Wolaita Sodo University Comprehensive Specialized Hospital
